# Some Advantages of a Swaged Shell Crown

**Published:** 1904-02-15

**Authors:** W. A. Knowles


### SOME ADVANTAGES OF A SWAGED SHELL CROWN.

BY W. A. KNOWLES, M.D., D.D.S.

Read before the San Francisco Dental Association.

Before considering the swaged crown, a few words on crowns in general may not be entirely out of place.

There are many points to be taken into consideration in the operation of covering a tooth or root with a shell crown.

If the tooth selected for crowning is in articulation with the teeth of the opposite jaw, it must be sufficiently reduced to allow for the thickness of the metal shell which is to assume the articulation.

If a root is selected for crowning, it should first be placed in a healthy and aseptic condition, being built up to the proper shape for giving the abutment the greatest strength.

The materials to be used for thus strengthening the weak root must be carefully considered.

If the walls are weak and need bracing, a sticky cement may be used to build the root to proper contour, but this material is contra-indicated when the filling should of necessity be placed below the gum level, because the acid secretions would be apt to attack it at this point, whenever the least recession of the gum tissues would allow the fluids of the mouth to come in contact with the cement filling. Cement will give great strength when fully protected, but otherwise its use is inadvisable.

Amalgam makes a splendid material for use under conditions which would exclude the secretion of cement. The secretions, were it exposed to them, would have little effect on it, and the film of cement which is left between the amalgam and the metallic shell would prevent the contained mercury attacking it, or in a manner acting deleteriously upon it.

In many cases, red base-plate gutta-percha makes an excellent filling material, especially when the walls left standing are fairly well preserved.

In every case, where a tooth or root has been prepared for a shell crown, before setting the crown the surfaces to be covered should be cleaned by means of a thoroughly efficient antiseptic. This gives greater chance of permanency to the operation and is very pleasing to many patients.

It is needless to say that all full crowns should extend under the gum margins as far as they can be carried with comfort, allowing for some recession which, as time goes on, will usually occur.

In considering assembled crowns, or those made in sections, which are afterward joined together, we find that there are many methods of procedure.

In making the band a butted joint is advantageous in some ways, but is not as strong as a lapped band.

A lapped joint has greater strength but is less accurate in the fit, there being a tiny spot which stands off at the root line, but which in practice may make but little difference.

A joint formed by butting the ends of the band, using no solder but sweating it into a continuous band is good, but requires considerable practice to successfully accomplish.

A seamless band drawn from a disk is best, and is easily made.

The best assembled crown is made by the use of a seamless band, to which a top has been soldered in proper position.

The best shell crown that can be made, however, is one which is constructed from a single piece, of even thickness throughout and accurately fitted to the margins, the proximal contour and the articulation.

The process of swaging from within appears to be faulty, inasmuch as it has a tendency to stretch the metal, rendering it very thin at points and correspondingly weak.

The swaging from without inward is best, because it maintains an even thickness of material and brings all surplus to the open end of the capsule or cartridge, much in the same manner as one would properly place a glove upon the fingers. If the proper measurements have been accurately taken, the crown swaged in this manner will fit absolutely and can be depended upon. It is compression instead of expansion.

Should an error have in any manner been made, it can much more readily be rectified in a crown which has been manufactured by this process.

The diameter of the free end may be enlarged or reduced by proper contouring appliances.

If the articulation is too heavy, it is not impossible to cause the crown to be bitten into proper position, by first annealing it so as to render it as malleable as possible, and then replacing it upon the tooth and directing the patient to close the teeth upon it as forcibly as possible.

Another method of correction is to fill the shell full of modeling composition and when cool, with a hard mallet and smooth point plugger, tap into proper position for articulating with its opponent.

If it should still be unsatisfactory, the model upon which it was made may be trimmed into proper shape and a new crown swaged in a few moments. If the trial band is too short to reach under the gums at any point, allowance may be made in the model, which will cause the completed crown in gold to reach to the position desired.

The swaged crown is the only one by means of which a skin fit may be obtained, and it is a well-known fact that the nearer the crown is in apposition to the root, the less cement will be necessary and the greater strength will be obtained in its attachment.

A swaged crown makes the best open-faced variety, and all swaged crowns have the advantage that they can be re-enforced externally as well as internally with less danger of changing shape when put under heat.

They may be soldered into bridges with much less liability of accidental melting.

There is not so much waste in material, and every bit may be used over again, there being no solder contamination.

No solder need to be used on the swaged crown until it has been accurately fitted to the tooth or root, after which it may be re-enforced as desired.

The objection to a swaged crown is the greater length of time necessarily consumed in the process of manufacture.

It can, however, be taken up now and then, a few moments at a time, and other operations simultaneously carried on.

It has been urged that it is much more economical to send the models to the plate-worker and allow him to perform the work at a much less cost than the value of the time when one does the work himself.

The objection to this is that you are not always able to depend upon the purity of the alloy used, and some crowns have been turned out that would cause tissue paper to blush, were it capable of doing so.

A molar crown, re-enforced, should weigh on an average twenty-four grains, but some have been weighed and found to contain but nine grains.

Crowns should be of about No. 30 thickness, never less, and the alloy of the material may be varied to suit the requirements of the case in hand. They should never be below 22k., and where strength and richness of color is desired there should be some copper in the alloy as well as silver. It is even possible to use 23¾k. gold successfully. The admixture of gold and silver, leaving out the copper, makes a much softer alloy, but is more "brassy" in color. Pure gold is too soft to withstand mastication. Crown metal, or platinum with a gold surface, has advantages. It is soft, will not melt under fire, but is expensive, inasmuch as the scraps are not very easily refined, and, in case of wear upon them, might show the platinum color where the gold should become removed.

Platinum crowns have been used in porcelain work, and at other times have had gold solder flowed over them.

In doing swaged crown work we should, at odd moments, make up a large number of blank capsules of various sizes and a crown may then be much more rapidly fashioned when required.

If one makes his own alloys, he should melt from forty to sixty dollars' worth at a time, for in large amounts there is liable to be greater accuracy in the proper admixture of the component metals.

It is also well to number all of the molds made for the various cases, keeping a list of each one, so that in case of necessity a new crown may be quickly and inexpensively made.

Several times patients have had crowns become loosened on account of the cement failing to hold, and in two cases the crowns were pulled off by eating sticky candy and were bitten flat together before the patient was aware of the accidental removal. In such a case it may be very difficult or even impossible to again restore the crown to proper shape, and, if the model had been retained and numbered, a new one could be quickly constructed.

In making these molds for preservation, a little copper former may be used for pouring the plaster into, which will give neatness, uniformity and least waste of time and material in dressing into shape.

Sometimes it becomes advisable to use a gold dummy in bridge or plate-work, and if such is the case, one can go to his numbered models, select one of proper shape and size and quickly manufacture it.

It is seldom that a crown made for one person will accurately fit another root in a different mouth, but such cases have been met with, and where one was a misfit a crown made for another was called into requistion and a second one again made in place of the one substituted.

The first crown ever made by the writer was from a published description of the operation, none having at that time been seen by him. It was worn for about twenty years with comfort.

Shell crowns are in many cases a blessing. They may be used to resurrect old roots, otherwise useless snags, and to restore split teeth to usefulness, but from an artistic standpoint they are often misused and are placed in locations where good judgment would contra-indicate their use. If you expect permanency, do not articulate a gold crown against an artificial tooth. If you desire to enlarge a crown at the cervical edge it is better to anneal it before attempting to do so, as in a swaged crown which has been tempered in the beating which it has received, there is some liability to splitting the gold by so doing. If it fits too snugly in any case, annealing it will cause it to become much more readily adapted to the shape of the root.—*Pacific Gazette.*

